# Efficacy of 2% ibuprofen subgingival irrigation as an adjunct to non-surgical therapy in the treatment of chronic periodontitis: A randomized controlled, split-mouth, clinical trial

**DOI:** 10.15171/japid.2019.012

**Published:** 2019-12-18

**Authors:** Amirhossein Farahmand, Ferena Sayar, Zohreh Omidali, Mahsa Soleimani, Bahareh Jafarzadeh Esfahani

**Affiliations:** ^1^Department of Periodontics, Faculty of Dentistry, Islamic Azad University, Tehran, Iran; ^2^Department of Periodontics, Tehran Medical Sciences, Islamic Azad University, Tehran, Iran; ^3^Dentist, Private Practice

**Keywords:** Chronic periodontitis, ibuprofen, irrigation, non-surgical, periodontal therapy

## Abstract

**Background:**

Pharmacological factors, such as ibuprofen, released topically in the periodontal pocket modulate the host response and enhance the influence of non-surgical periodontal treatment.

**Methods:**

In this double-blind, randomized, split-mouth, clinical trial, 38 outpatients with mild to moderate chronic periodontitis were enrolled by applying the simple random sampling method. They had at least one tooth with a periodontal pocket depth of >4 mm in each quadrant and had undergone phase I of periodontal treatment one week after scaling and root planing (SRP). The parameters of clinical periodontal evaluation, including probing pocket depth (PPD), clinical attachment level (CAL), plaque index (PI), and bleeding index (BI), were measured. In addition, two mandibular molar teeth in one quadrant were randomly nominated for subgingival irrigation with 0.5 mL of 2% ibuprofen or placebo mouthwash. The measurements were repeated after at least one week for three months.

**Results:**

Thirty-four individuals (18 women and 16 men), with an age range of 28‒36 years, were evaluated for three months. Moreover, periodontal clinical parameters were assessed within three months. There was a significant improvement in pocket depth (PD) and clinical attachment level (CAL) readings after 12 weeks in both groups (paired t-test). On comparing, the group with scaling and root planing (SRP) + ibuprofen showed more favorable results than the group with SRP + placebo (P<0.05). There were significant improvements in PI and BI in both groups; the differences between the two groups were significant (P<0.05).

**Conclusion:**

The mouthwashes containing ibuprofen might reduce the symptoms of periodontal disease and might be used as an adjunct in the healing process.

## Introduction

Chronic periodontitis is an infectious disease marked by periodontal pocket formation that results in the inflammation and destruction of periodontal tissues.^1^ It is well known that periodontal disease mainly progresses due to bacterial infection; however, the initiation and progression of the disease can vary among objects in terms of genetic predisposition, systemic status, and environmental effects.^2-4^ There is a large body of studies showing that both surgical and non-surgical periodontal treatments are effective against periodontitis by eliminating pathogenic dental plaque and calculus.^5^ Non-surgical periodontal treatment reduces pocket depth (PD) and increases clinical attachment level (CAL) to some extent;^6,7^ however, it cannot fill the bony defect.^8^ In addition, some patients do not respond to traditional periodontal treatment,^9^ or exhibit a highly elevated susceptibility to periodontal disease.^10^ Assessment of the mechanisms underlying these events has shown that the immune response of patients seems to play a critical role in the extension and the manifestation of periodontal diseases.^11,12^ The host modulation treatment is to restore the balance of pro-inflammatory or destructive mediators and anti-inflammatory or protective mediators to that seen in healthy patients. Host modulation treatment is a method believed to decrease tissue damage and maintain or even restore inflammatory tissues by altering host response agents.^13^ However, non-steroidal anti-inflammatory drugs (NSAIDs) are known as inhibitors of the formation of Prostanoids (including prostaglandins and thromboxane), and this has been the source of much interest in the inhibitors of the host immune response to periodontal disease. Prostanoids are produced during the activation of the cyclooxygenase pathway to periodontal disease, which is associated with tissue destruction and bone loss. Investigators suggest that selective NSAIDs (COX-2 inhibitors) might reduce the bone loss associated with periodontitis;^14^ additionally, host modulation using various therapeutic agents targeting the manipulation of the inflammatory pathway has been proposed as an adjunctive treatment with conventional periodontal therapy.^15-18^ The latest survey determined that the use of NSAIDs in combination with mechanical periodontal therapy improved bone maintenance in treating patients with periodontal diseases.^19^ Of all the various combinations of these, such as flurbiprofen, ibuprofen is readily absorbed through the gingival tissues. Moreover, the development of local NSAID formulations (e.g., gels, toothpaste, and rinses) with daily employment appears to be of particular interest. These products might result in a greater decrease in the harmful systemic influences of non-selective NSAIDs in the long-term host modulation of periodontitis-susceptible subjects (20). Furthermore, studies have shown that prostaglandin production inhibitors, including non-steroidal anti-inflammatory drugs (NSAIDs), can influence this phase of bone loss in periodontal disease.^21^ The current study aimed to evaluate the clinical effectiveness of subgingival irrigation with 2% ibuprofen as an adjunct to scaling and root planing (SRP) in patients with chronic periodontitis.

## Methods

### Study Protocol and Selection of Patients

This study was carried out as a single-center, examiner-blinded, randomized, split-mouth clinical trial, with a two-arm, parallel-group design, in three months. This study was undertaken to assess the clinical application of a combination plus locally applied ibuprofen (2%) mouthwash (made in the Faculty of Pharmaceutical Sciences, Islamic Azad University, Tehran, Iran) in combination with SRP versus SRP + placebo mouthwash. The clinical measures of periodontal disease were evaluated in patients attending the Department of Periodontics, Faculty of Dentistry, Islamic Azad University, Tehran, Iran. Thirty-eight patients of both genders, aged 28‒35 years, were selected, who were diagnosed with mild to moderate chronic periodontitis conforming to the 1999 Classification of Periodontal Diseases and Conditions with a probing depth >4 mm (Academy of Periodontology in 1999).^22^

### Inclusion Criteria

The inclusion criteria consisted of a confirmed diagnosis of early moderate chronic periodontitis, at least two residual areas with a probing pocket depth (PPD) of >4 mm in two opposite quadrants, and CAL of ≥1‒2 mm, at least 20 remaining teeth with two teeth in every mandibular quadrant. The clinical periodontal parameters were determined and recorded at baseline within seven days for 12 weeks in the selected teeth, at six locations in each tooth, using a periodontal probe (Williams Probe, Hu-Friedy, USA) by two calibrated masked investigators (periodontists). Pocket depths were defined as the distance from the gingival margin to the bottom of the pocket, and CAL was defined as the distance from the cementoenamel junction (CEJ) to the base of the pocket. PI was measured using a Silness & Löe index.^23,24^ BoP was evaluated through visual inspection 30 seconds after probing according to Carter & Barnes (score 0: no bleeding after probing; score 1: a single separate bleeding point becomes visible after probing).^25^

### Exclusion Criteria

Patients with the following conditions were excluded: known hypersensitivity to the components of the formulation, those with systemic disease, pregnant and breastfeeding women, those undergoing orthodontic treatment, those with numerous dental bridges, those taking anti-inflammatory drugs, antibiotics, or immunosuppressive medicines in the latest three months, those wearing a partial denture, those with numerous carious lesions, those habitually smoking, and those with a history of periodontal surgery.

### Intervention

The periodontal clinical parameters were noted at the baseline. All the subjects received full-mouth SRP and polishing at baseline. Oral care instructions were given that included tooth brushing for two minutes twice a day with the modified Bass technique with a soft-bristled brush (soft toothbrushes protect gums and gingival margin against damage). Moreover, the subjects were randomly assigned to two groups of equal size. Group A received SRP combined with ibuprofen mouthwash; group B subjects were treated with SRP in combination with a placebo mouthwash. The periodontal pocket depth was defined at six areas per tooth; also, the left and right mandibular quadrants were allocated. In both categories, the mouthwashes (2% ibuprofen or placebo) were applied to all the periodontal pockets (pocket depth of >4 mm), using an insulin syringe with the blunt needle inserted into the bottom of the periodontal pocket; 0.5 mL of 2% ibuprofen mouth rinse was applied to each surface (distal, mid, and mesial) of the lower first and second molars. Then, all the pre- and post-treatment periodontal clinical parameters were registered by two investigators who were masked to the type of medication, while another clinician delivered the respective operations to both groups. The patients were recalled at 7-day intervals within three months to examine periodontal clinical parameters.

### Sample Size

Conforming to previous examinations, the sample size evaluation was based on identifying changes in the principal consequences of periodontal disease measurements ‒ probing depth (PD) from the baseline to the end of the follow-up. The sample size of the study was calculated using “two-sample t-test sample size calculation” tab of MINITAB software, considering α=0.05, β=0.05, a mean difference of 1.65, and pooled standard deviation=1.4. Finally, 17 subjects were necessary for each group.

### Randomization and Blinding

Randomization was carried out by one of the investigators who did not have a role in the treatment of the subjects. Eligible participants who proved their ongoing commitment to the study were block-randomized to treatment in a double-blinded manner, implemented as block randomization with a 4:1 allocation. All the mouthwashes were placed in identical bottles and labeled A or B. Each participant picked one symbol from a box to decide his/her sequence of the use of mouthwashes. The randomized order of mouthwash use during the entire clinical study was controlled by a calibrated examiner. Moreover, before initiating the investigation, an examiner (a periodontist) was trained in a calibration process twice until it reduced the researcher reliability. The examiner was calibrated for one week before the start of the experiments. Periodontal assessments were performed by a single calibrated examiner. The examiner evaluated the subjects on two occasions: at baseline and after two days. Calibration was validated, as 90% of the readings were reproduced within a 1.0-mm difference.

### Statistical Analysis

SPSS 21.0 was used for the analysis of data. Student’s t-test was applied for intragroup comparisons of the paired samples. ANOVA was employed for intergroup comparison using the mean variance values of the clinical periodontal parameters (plaque index or bleeding scores) on the first day to the last appointment within three months. P-values<0.05 were considered as statistically significant.

## Results

**Figure 1:**
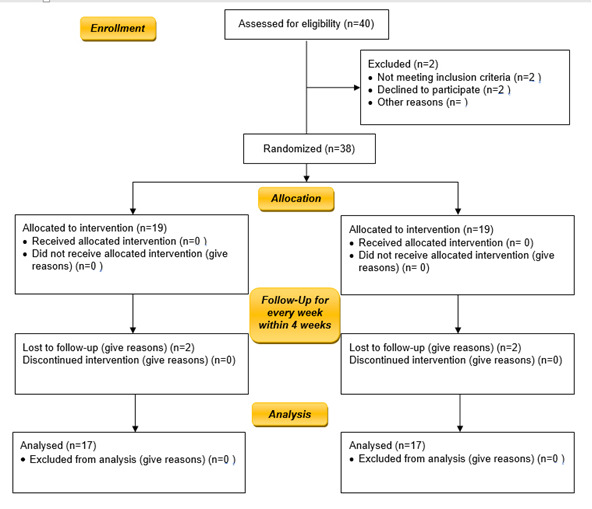
CONSORT 2010 Flow Diagram

The mean age of the subjects was 28‒35 (31.0±3.2) years in both groups. Gender distribution included 18 women and 16 men in the two groups (Figure 1). The initial analysis evaluated intention-to-treat and involved all the subjects. Two patients did not record back for the first follow-up appointment, and two patients from each group failed to return for follow-up visits after the second visit. Therefore, they were excluded from the current study. At baseline, no significant differentiation was observed between the groups in any of the clinical periodontal parameters. After thorough SRP, followed by routine maintenance, the rate of plaque control in all the patients proved adequate; during the examination, plaque development decreased with significant differences between the two groups. Also, dental plaque deposits were minimal, with significant differences between the two groups. Statistically, a significant difference was found in PI between the ibuprofen and placebo mouthwashes after 12 weeks. However, the PI score was significantly higher in the ibuprofen mouthwash group compared to the placebo group at the three-month interval (P<0.05) (Table 1). The BI and PD significantly decreased in the ibuprofen group compared to the placebo group after 6 and 12 weeks (P<0.05; Table 2 & Table 3). However, the CAL was significantly different only after 12 weeks in the ibuprofen group compared to the placebo group (P<0.05) (Table 4). The data were further analyzed to define variations in the distribution of the CAL before and after treatment. The ibuprofen group exhibited a significantly higher CAL than the placebo group. In the ibuprofen group, the percentage of patients with a gain in CAL between 1.5 and 2 mm was 0% versus 17% at the 12-week interval, indicating an improved CAL in the ibuprofen group compared to the placebo group. All the subjects tolerated the medicine well without any complications or adverse reactions. The soft tissues healed within normal limits, and no significant differences were observed.

**Table 1:** The effect of both treatments on the mean values of plaque index (PI) among the study groups at baseline, 0, 1 and 3 months after treatment

**Plaque index**	**Ibuprofen group**	**Placebo group**
		
**Baseline**	0.1±0.73	0.1± 0.74
**6 weeks**	0.43±0.03	0.49±0.02
**12weeks**	0.38±0.01	0.43±0.02
**p.value**	P<0.0001

**Table 2:** The effect of both treatments on the mean values of Bleeding index (BI) among the study groups at baseline, 0, 1 and 3 months after treatment

**Bleeding on probing**	**Ibuprofen group**	**Placebo group**
**Baseline**	0.97±0.68	0.98±0.67
**6 weeks**	0.63±0.05	0.85±0.09
**12weeks**	0.50± 0.06	0.73±0.08
**P-value**	P<0.0001

**Table 3:** The effect of both treatments on the mean values of pocket depth (PD) among the study groups at baseline, 0, 1 and 3 months after treatment

**Pocket depth**	**Ibuprofen group**	**Placebo group**
**Baseline**	4.60±0.86	4.61± 0.83
**6 weeks**	3.40±0.15	3.86 ±0.15
**12weeks**	2.70± 0.18	3.32±0.21
**P-value**	P<0.0001

**Table 4:** The effect of both treatments on the mean values of Clinical attachment level (CAL) among the study groups at baseline, 0, 1 and 3 months after treatment

**CAL**	**Ibuprofen group**	**Placebo group**
**Baseline**	4.82±0.49	4.82±0.51
**6 weeks**	3.56±0.10	3.92±0. 12
**12weeks**	2.90±0.16	3.50 ±0.23
**P-value**	P<0.0001

## Discussion

The present clinical study showed that COX-1 inhibitors might significantly decrease periodontal diseases and the healing of periodontal parameters compared to placebo mouthwash. On the other hand, the results showed the clinical efficacy of 2% ibuprofen mouthwash as an adjunct to periodontal debridement for the treatment of periodontal diseases. Furthermore, several studies have indicated the effect of the host response on the periodontium by blocking and modulating periodontal disease development.^26^ In the present study, the changes in periodontal parameters after subgingival irrigation with ibuprofen led to a significant reduction in bleeding on probing at baseline and after 15, 30, 45, 60, 75, and 90 days.

Consistent with the present study, Yewey et al^27^ showed in beagle dogs that the application of a biodegradable subgingival delivery system for flurbiprofen resulted in a significant reduction in gingival inflammation. In another study, it was shown the statistically significant recovery in the gingival situation of the subjects with the use of a flurbiprofen gel as an adjunct to SRP as correlated with oral prophylaxis or flurbiprofen gel only.^28^ Paolantonio reported that subgingival irrigation with 1% ASA in patients with periodontal disease resulted in a reduction in subclinical inflammation of the periodontal pockets.^29^ Paquette et al^30^ showed that at the 90-day interval, the reduction in BoP was highly significant compared with the two other groups.

Srinivas et al^31^ reported the most considerable decrease in BoP with ketoprofen, which was significant, but it was found to be ineffective in reducing the pocket depth and CAL when ketoprofen gel was used. Vogel et al^32^ showed that the local steroidal drug significantly prevented gingival inflammation, whereas the systematically applied non-steroidal drug had no apparent effect, also concluding that there was no significant effect on gingival crevicular fluid and BoP. Furthermore, Corry and Moran^33^ suggested the use of strips of polymethacrylate cement as a delivery vehicle for the sustained release of NSAIDs, such as indomethacin, as an essential system for treating periodontal diseases.^33^ However, the decrease in gingival inflammation might be attributed to the lowered plaque toxicity, interference with the subgingival plaque maturation, or possibly washing away unattached plaque.^34-36^

Furthermore, the present study showed a decrease in the clinical parameters of probing depth and clinical attachment levels. Del Puente et al^37^ showed that the use of NSAIDs in cases with adult periodontitis resulted in less attachment loss. Funosas et al^38^ showed that intra-crevicular application of 1% ASA and 2% ketoprofen gel as an adjunct to periodontal treatment in patients with chronic periodontitis could significantly reduce probing depths. In another study, the maximum PD reduction was observed in a few deep sites (7 mm) in the SRP‒loxoprofen group compared to the SRP‒placebo group.^39^ However, several studies have shown that subgingival irrigation decreased mean probing depths by only one mm.^40,41^ Nonetheless, several studies exhibited an even less reduction.^42-46^ In other words, In root planing with irrigation therapy, probing pocket depths decreased up to 2 to 3 mm.^44,47-49^ Therefore, if probing pocket depth decrease is required, root planing is indicated.

Furthermore, different agents that might influence drug delivery, such as calculus, irrigator head design, and irrigation pressure, have also been investigated.^50^ The present study also showed a significant change in the plaque index score at 90 days. On the contrary, Johanson et al^51^ showed that the drug has no significant effect on the plaque or bleeding index scores. Naprosyn might increase healing following the removal of microbial plaque; however, this drug does not suppress the inflammation-inducing properties of plaque, Naprosyn might increase recovery following plaque removal.^51^ It is noteworthy that subgingival irrigation with medicines decreased plaque indices but failed to reduce the symptoms of inflammation;^48-55^ however, when root planing was also employed, there were fewer bleeding spots.^44,49,50,56,57^ Although the reduction in the inflammation of gingiva might have been due to lowered plaque toxicity, interference with the subgingival plaque maturation, or possibly removal of unattached plaque.^34-38^

Farahmand et al^58^ reported that the Ibuprofen gel, as an adjunct to SRP, might open up new horizons in the management of periodontal therapy and could be used to supplement the treatment to determine the inflammatory process and clinical signs of the disease more rapidly. Batool et al^59^ injected 1.5% chlorhexidine and ibuprofen into the periodontal pockets in an experimental periodontitis mouse model and reported a reduction in inflammation and an improvement in periodontal wound healing through inflammatory cell scoring after treatment. Furthermore, another study showed that electrospun polycaprolactone scaffold functionalized with ibuprofen significantly improved the clinical attachment, reduced inflammation-induced bone loss, efficiently and differentially controlled inflammatory and migratory gingival cell reaction, and probably promoted periodontal regeneration.^60^ That is why topical use is a valid alternative for completing the treatment of periodontal disease.

Local forms used in dentistry involve mouthrinses, supra- and subgingival irrigation, and subgingival devices. Subgingival or intra-crevicular tools improve the time that the active element remains within the periodontal pocket, thus assuring a longer delivery time. Various subgingival devices, as vehicles for delivering NSAIDs, have been studied, such as the subgingival irritation tools described by Paolantonio et al.^29^

By improving oral hygiene habits plus periodontal therapy, a mechanical plaque control treatment on bacterial plaque could be reached, after which the anti-inflammatory action of NSAIDs could be applied in perfect treatment during a healing process. Furthermore, as there are many inflammatory mediators correlated with the host response in periodontal disease, it is necessary to clarify what drug offers the several appropriate effects on bone, soft tissues, and even upon some inflammatory mediators. However, the topically administered NSAIDs protocols employed in this investigation did not alter the plaque index more than the placebo did.

As the infectious factors that produce and improve the inflammatory process need to be reduced to enable NSAID anti-inflammatory activity, the plaque score might be expected to be a variable independent of NSAIDs employment, and more related to mechanical debridement, basic therapy, or changes in the patient's brushing method. However, it should be pointed out that some primary data imply that irrigation with high concentrations of substantive drugs might enhance the efficiency of root planing. Therefore, further research is needed to show the efficacy of this treatment modification. Hence, subgingival irrigation might be useful when root planing is less effective due to anatomical or other factors. Nevertheless, it becomes evident that the major failure of irrigation treatment is the immediate removal of subgingivally placed medications.

## Conclusion

Mechanical periodontal therapy is the most effective procedure for the treatment of chronic periodontitis. Now, it seems the use of topical medications might reduce the manifestations of periodontal disease, as well as mouthwashes containing ibuprofen.

## Authors’ Contributions

AF and MS designed and performed the experiments AF and FS conceived of the idea. AF, FS, and ZO implemented the study and performed data analysis. AF and BJE prepared the manuscript. MS edited the manuscript.

## Competing Interests

The authors declare no conflict(s) of interest related to the publication of this work.

## Ethics Approval

The research protocol was reviewed and approved by the Ethics Committee (code of ethics: 2161) of the Faculty of Dentistry, and also registered in the Iranian Registry of Clinical Trials (code: IRCT2014050917587N4) and the clinicaltrials.gov (code: NCT02538237). The study procedure conforms to the principles outlined in the Declaration of Helsinki on human medical experimentation. Verbal and written informed consent was obtained from all the participants.
